# Evolving Guidelines in Pericarditis: Aligning Practice With Pathophysiology

**DOI:** 10.1016/j.jacadv.2025.102509

**Published:** 2026-01-28

**Authors:** Muhammad Ehsan, Jibran Ikram, Joseph El Roumi, Syed Waqas Haider, Paul C. Cremer, Tom Kai Ming Wang, Allan Klein

**Affiliations:** a Center for the Diagnosis and Treatment of Pericardial Diseases, Section of Cardiovascular Imaging, Department of Cardiovascular Medicine, Heart, Vascular and Thoracic Institute, Cleveland, Ohio, USA; b Pericardial Disease Program, MedStar Heart and Vascular Institute, MedStar Georgetown University Hospital, Washington, District of Columbia, USA; c Bluhm Cardiovascular Institute, Northwestern Medicine, Northwestern University Feinberg School of Medicine, USA

**Keywords:** echocardiography, heart failure progression, hs-cTnT, NT-proBNP

Pericarditis management has long been hindered by high recurrence rates, a one-size-fits-all reliance on corticosteroids, and fragmented care. Clinicians have navigated complex cases without a clear compass, often relying on consensus-based protocols that lagged advances in pathophysiology, imaging, and targeted therapy.

Pericarditis has a long-recognized history in medicine, with its earliest descriptions dating back to ancient times, but systematic clinical recognition began in the 19th century. The 2004 European Society of Cardiology (ESC) pericardial guidelines^1^ were the first systematic attempt to standardize care, followed by the 2013 American Society of Echocardiography imaging guidelines^2^ emphasizing the role of echocardiography in the management of pericardial disease, while recognizing the emerging role of multimodality imaging including cardiac magnetic resonance (CMR). The 2015 ESC guidelines laid the foundation for the classification and management of pericardial diseases. As understanding of recurrent pericarditis has evolved, particularly regarding its autoinflammatory pathophysiology and the effectiveness of interleukin (IL)-1 inhibitors, the solid foundation laid by the 2015 ESC guidelines has begun to show its limitations.^3^ The 2025 American College of Cardiology (ACC) concise clinical guidance document builds on the 2024 international position statement^4^ and represents a welcome advance, prioritizing a precision, phenotype-driven model that leverages advances in pathophysiology, imaging, and targeted management^5^ (Figure 1). Shortly thereafter, the ESC released its 2025 guidelines, which recognized the growing role of advanced imaging and biologic therapies in the management of recurrent pericarditis but ultimately retained the traditional stepwise treatment algorithm outlined in the ESC 2015 guidelines.^6^ This stands in stark contrast to the ACC’s modernized framework. This commentary critically examines these updates, contrasts them with the 2015 and 2025 ESC guidelines, and explores implications for clinical practice.

**Figure 1:**
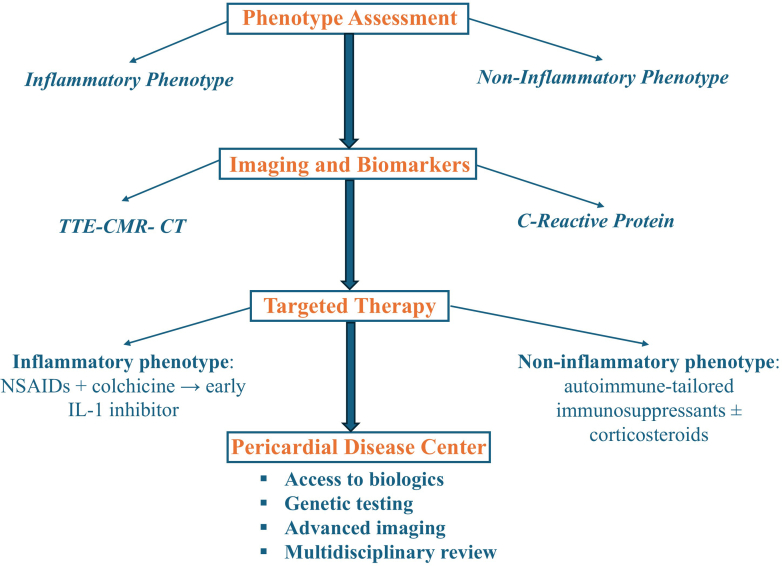
Precision, Phenotype-Driven Management of Pericarditis: The 2025 ACC Guidance Framework Precision, Phenotype-Driven Management of Pericarditis: The 2025 ACC Guidance Framework. ACC = American College of Cardiology; CMR = cardiac magnetic resonance; CT = computed tomography; NSAID = nonsteroidal anti-inflammatory drug; TTE = transthoracic echocardiogram.

The 2015 ESC guidelines set the groundwork for pericarditis management, recommending nonsteroidal anti-inflammatory drugs and colchicine as first-line therapy, with corticosteroids used as second-line therapy, and immunosuppressive agents (eg, azathioprine, intravenous immunoglobulin, anakinra) as third-line options for colchicine-resistant, corticosteroid-dependent recurrent pericarditis.^3^ However, these recommendations were based mostly on expert consensus, with minimal reliance on advanced multimodality imaging or phenotypic stratification. Since their publication, the landscape has shifted, with emerging evidence focusing on autoinflammatory biology in recurrent pericarditis and the role of biologics.^4^

The 2025 ACC document builds on this foundation by clearly endorsing a phenotype-driven approach, utilizing advanced imaging (eg, CMR) and biomarkers (eg, C-reactive protein) to guide therapy selection and duration.^5^ ACC 2025 modernizes diagnostic criteria by replacing the 2015 ESC “2 of 4” criteria with pericarditic chest pain plus at least one of the following: pericardial friction rub, typical electrocardiogram changes, new or worsening pericardial effusion, elevated inflammatory markers, or CMR/computed tomography (CT) evidence of pericardial inflammation. This elevates biomarkers and CMR from supportive to central elements of diagnosis, aligning with contemporary disease biology. Both the ESC and ACC documents introduced a tiered diagnostic approach (definite, possible, unlikely) for pericarditis; however, the 2025 ESC guidelines allow for atypical presentations in the absence of classic chest pain.^6^

While echocardiography remains the first-line diagnostic modality, the ACC and ESC expand the role of CMR and CT for both diagnostic and therapeutic planning for recurrent pericarditis. CMR is now considered vital for detecting pericardial inflammation using late gadolinium enhancement and and T2-weighted imaging for pericardial edema, as well as for assessing constriction and risk stratification. CT holds significant diagnostic value in constrictive pericarditis as it can identify pericardial thickening, calcification, and other structural changes. These techniques guide risk assessment and treatment selection.^5^

Therapeutic strategy has shifted from stepwise escalation to phenotype-driven care. IL-1 inhibitors (rilonacept, anakinra) are now recommended as the second-line therapy by the ACC for patients with multiple recurrences who are colchicine resistant, especially those with an autoinflammatory phenotype (pericardial inflammation evidenced by elevated inflammatory markers or findings on CMR).^5^ The ESC 2025 guideline also embraces the role of IL-1 inhibitors by granting them a Class 2 recommendation.^6^ However, it preserves the conventional management algorithm, in direct contrast to the modern approach adopted by the ACC, largely due to differences in pharmacoeconomics and the availability of IL-1 inhibitors in Europe.

This change is reinforced by several clinical studies^7^ such as the RHAPSODY (Phase 3 Trial of Interleukin-1 Trap Rilonacept in Recurrent Pericarditis) trial which found rilonacept to be effective in reducing recurrence rates, hastening symptom resolution, and enabling successful corticosteroid taper (7% vs 74% recurrence rates in the randomized withdrawal period). Rilonacept has been approved by the Food and Drug Administration for the management of recurrent pericarditis, with a recommended dosage of 320 mg subcutaneously on day 1 followed by 160 mg weekly; treatment duration should be individualized and guided by clinical, biomarker, and imaging remission. The AIRTRIP trial was a randomized withdrawal trial that endorsed anakinra’s efficacy in the management of colchicine-resistant, corticosteroid-dependent patients with recurrent pericarditis (18% vs 90% recurrence rates in the anakinra vs placebo groups respectively after withdrawal). Goflikicept, another biologic, was also found to be efficacious in the management of recurrent pericarditis. Furthermore, various observational studies and large registries endorsed the efficacy and safety of IL-1 inhibitors, with the most adverse events being mild (injection site reactions or upper respiratory tract infections). These drugs are also associated with significant improvements in quality-of-life outcomes as reported in the RHAPSODY and AIRTRIP trials. This stands in stark contrast to the myriads of adverse events associated with long-term corticosteroid use as a second-line option according to the prior and current ESC guidelines. Nevertheless, corticosteroids are still reserved for noninflammatory phenotypes or autoimmune-related pericarditis. Together, these studies validated the paradigm shift in the management of recurrent pericarditis, a direction embraced by the ACC 2025 but only cautiously acknowledged by the ESC 2025 guidelines.

By preserving corticosteroids as the standard second-line therapy, the ESC 2025 guideline risks perpetuating the very recurrences, metabolic complications, and dependency that the modern evidence base seeks to overcome.^6^ However, this approach is more pragmatic considering the availability and cost associated with IL-1 inhibitors. In contrast, the ACC 2025 guidance reflects a decisive modernization, one that finally aligns practice with pathophysiology.

The ACC advances the management of pericardial effusion by moving beyond simple volume thresholds to an integrated, imaging-based, and hemodynamic assessment. CT and CMR, owing to their ability to characterize effusion etiology and detect associated inflammation, are being used to guide intervention alongside echocardiography. Cardiac tamponade has been reframed as a dynamic process dependent on the rate of accumulation rather than absolute volume, highlighting the importance of coupling clinical features with imaging findings. This systematic approach ensures that pericardiocentesis is reserved for hemodynamic compromise, while inflammatory effusions can be managed medically. The ACC document stresses distinguishing transient inflammatory constriction from chronic calcific disease. With the use of advanced imaging, anti-inflammatory therapy could treat transient inflammation, while pericardiectomy is reserved for chronic, fibrotic cases. This imaging-driven classification also recognizes effusive-constrictive pericarditis as a distinct phenotype. This phenotypic approach, consistent with the broader theme of the guidance, reduces unnecessary surgery and aligns therapy with underlying biology.^5^

Another landmark shift is the formalization of pericardial disease centers and multidisciplinary teams recommended by the ACC and ESC documents respectively. These specialized, multidisciplinary centers, consisting of cardiologists, imaging specialists, and rheumatologists, may streamline management of pericarditis cases. By integrating imaging, access to biologics, and genetic testing, these centers have been associated with improved outcomes and more coordinated care.^8^

Despite these important advances, several challenges remain. The optimal duration of biologics remains unclear, as 75% of patients experienced a recurrence in the RHAPSODY extension after stopping rilonacept at 18 months.^9^ Additionally, the role of biologics in special populations such as patients with autoimmune disease, tuberculous pericarditis, effusive-constrictive pericarditis, or patients with a neoplastic etiology is uncertain due to the underrepresentation of these patients in trials. The high cost of IL-1 inhibitors and the limited availability of advanced imaging modalities, which form the cornerstone for guiding management, could disproportionately affect underserved populations. Additionally, a lack of physician familiarity with advanced imaging and novel therapies persists. Moreover, this highlights the need for dedicated U.S. guidelines on pericarditis, with regular updates every 3 to 5 years to reflect rapidly evolving evidence. Lastly, the impact of exercise restriction is still unclear, with limited data available to guide recommendations.

Pericarditis management is becoming increasingly personalized, with the incorporation of clinical, laboratory, and advanced imaging data to guide therapy selection and duration. Advanced CMR is being increasingly used for the stratification of disease severity and monitoring. Several ongoing trials are evaluating various other IL-1 blockers in managing recurrent pericarditis. The use of wearable devices to quantify physical activity and detect early signs of recurrence may refine exercise protocols in patients with pericarditis.

While ESC 2025 guidelines provide continuity, the 2025 ACC clinical guidance redefines pericarditis management, shifting from rigid algorithms to an individualized, phenotype-driven framework.^10^ Importantly, this is the first pericarditis management consensus document in the United States. By integrating multimodality imaging for diagnosis and risk stratification and targeting biologic therapy to inflammatory phenotypes, it offers an evidence-based pathway for tailored care. The evolution of pericarditis care will depend on the global adoption of the modern phenotype-driven model, which translates pathophysiologic insight into personalized treatment decisions.

## Funding support and author disclosures

Dr Klein has received grants from Kiniksa and Cardiol Therapeutics outside the submitted work and has served on scientific advisory boards for Kiniksa, Cardiol Therapeutics, Zydus, and Ventyx. Dr Haider has served on advisory board for Kiniksa. Dr Cremer’s disclosures are: Sobi, Norvartis, Kiniksa, CardiolRx, Pfizer, Boston Scientific, Ventyx, and Monta Rosa. All other authors have reported that they have no relationships relevant to the contents of this paper to disclose.
